# Fusing the single-excitation subspace with ${\mathbb C}^{2^n}$

**DOI:** 10.1038/s41598-020-79853-3

**Published:** 2021-01-11

**Authors:** Michael R. Geller

**Affiliations:** Center for Simulational Physics, University of Georgia, Athens, GA 30602 USA

**Keywords:** Quantum physics, Quantum simulation

## Abstract

There is a tremendous interest in developing practical applications for noisy intermediate-scale quantum processors without the overhead required by full error correction. Near-term quantum information processing is especially challenging within the standard gate model, as algorithms quickly lose fidelity as the problem size and circuit depth grow. This has lead to a number of non-gate-model approaches such as analog quantum simulation and quantum annealing. These come with specific hardware requirements that are different than that of a universal gate-based quantum computer. We have previously proposed an approach called the single-excitation subspace (SES) method, which uses a complete graph of superconducting qubits with tunable coupling. Without error correction the SES method is not scalable, but it offers several algorithmic components with constant depth, which is highly desirable for near-term use. The challenge of the SES method is that it requires a physical qubit for every basis state in the computer’s Hilbert space. This imposes exponentially large resource costs for algorithms using registers of ancillary qubits, as each ancilla would double the required graph size. Here we show how to circumvent this doubling by leaving the SES and fusing it with a multi-ancilla Hilbert space. Specifically, we implement the tensor product of an SES register holding “data” with one or more ancilla qubits, which are able to independently control arbitrary $n\!\times \!n$ unitary operations on the data in a constant number of steps. This enables a hybrid form of quantum computation where fast SES operations are performed on the data, traditional logic gates and measurements are performed on the ancillas, and controlled-unitaries act between. As example applications, we give ancilla-assisted SES implementations of quantum phase estimation and the quantum linear system solver of Harrow, Hassidim, and Lloyd.

## Introduction

The recent Google quantum supremacy experiment^1^ marks the beginning of an exciting era of quantum technology, where nascent quantum devices have advanced computational power and pose a challenge to the extended Church–Turing thesis. A key design feature of the Google experiment, which sampled from the outputs of random circuits, is the short circuit depths required, even for 53 qubits. A second feature is that it did not require high circuit fidelity, only a (statistically significant) nonzero value. However these features are atypical of most known quantum algorithms. While there has been a lot of effort dedicated to finding other short-depth applications, and expectation that some will be found^2^, achieving supercomputing power or beyond with noisy intermediate-scale quantum (NISQ) devices^3^, also called *prethreshold* devices^4^, appears to be extremely challenging.

The single-excitation subspace (SES) method^5–7^ is a non-gate-model approach that uses a complete (fully connected) graph of *n* superconducting qubits and performs quantum computations and simulations in the *n*-dimensional SES, where the device Hamiltonian is directly programmed. This eliminates the need to decompose operations into elementary one- and two-qubit gates, allowing larger computations to be performed with the available coherence time. Symmetric $n \! \times \! n$ unitaries can be implemented in a single fast step^6^, and nonsymmetric unitaries in three^7^. These building blocks lead to many algorithms with *O*(1) depth, which is highly desirable for NISQ applications. The method also enables quantum simulation of *n*-dimensional closed systems (for fixed evolution time) with constant depth^5,6^. We refer to the SES method as a non-gate-model approach because it cannot be implemented with a standard universal quantum computer, which lacks all-to-all tunable coupling.

Restriction to the SES means that a physical qubit is required for each basis state of the computational Hilbert space, and the method is not scalable. A technically unscalable architecture, however, might still be useful for practical NISQ computing^3^. But the scaling does impose large resource costs for algorithms involving ancillary qubits, as each ancilla would double the required graph size. Here we show how to circumvent this doubling by fusing the SES with a multi-ancilla Hilbert space, which necessitates leaving the SES. In particular, we show that a complete graph of $n+n'$ qubits can implement the tensor product of an *n*-qubit SES register holding “data” with $n'$ ancilla qubits, in such a way that each ancilla coherently controls the application of an arbitrary $n \! \times \! n$ unitary to the data. Crucially, the number of steps required to perform a set of $n'$ controlled-unitaries is *independent* of *n* and only linear in $n'$ (as the controlled-unitaries are performed serially).

**Figure 1:**
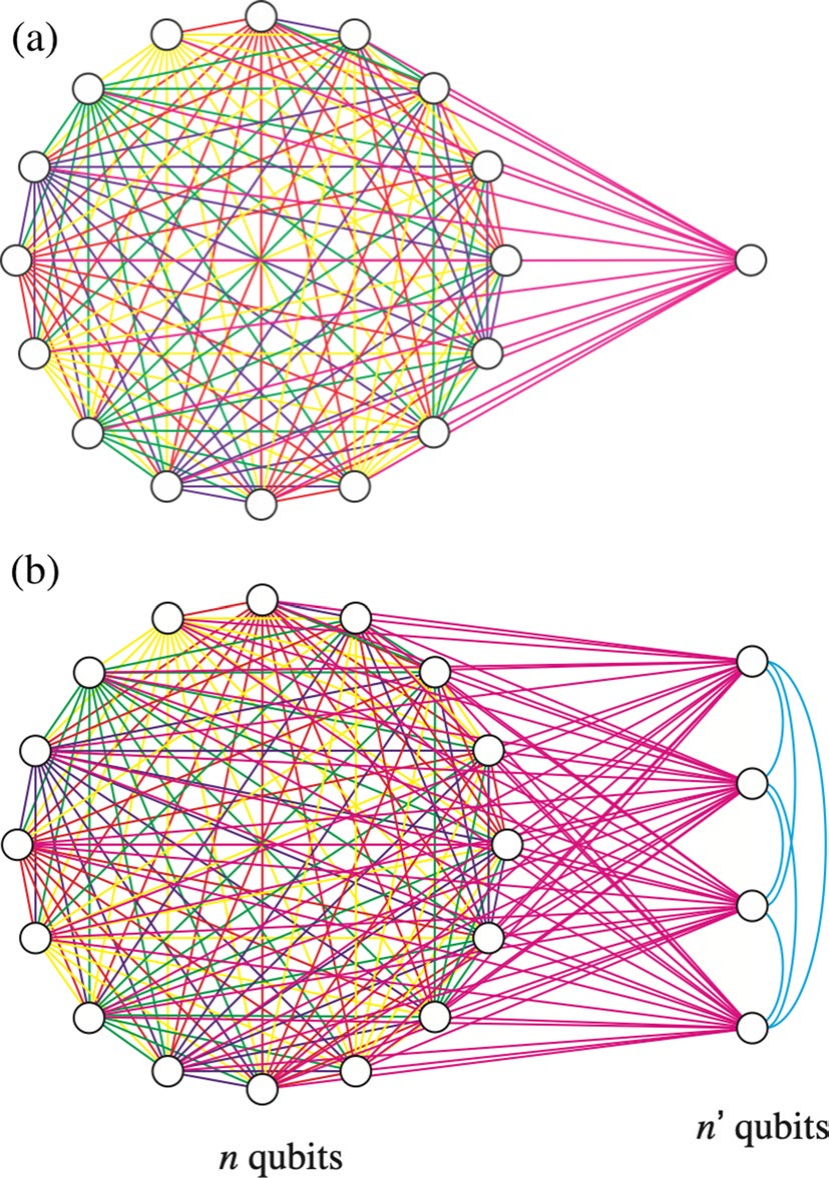
(**a**) Adding a qubit to the $n=16$ graph. (**b**) Adding a register of $n'$ ancilla qubits creates a hybrid form of SES and gate-based computation, where fast SES operations are performed on the data, traditional logic gates and measurements are performed on the ancillas, and controlled-unitaries operate between.

To better understand the tensor product structure consider adding a single superconducting qubit to an existing SES array, resulting in a complete graph of $n+1$ qubits; see Fig. 1a. There are two distinct ways of doing this, which we call *direct sum* and *tensor product*. The direct sum means that number of excitations remains unity and the dimension of the computational subspace is increased by one, resulting in an $n+1$-qubit SES register. Adding $n'$ qubits in this way increases the size of the register to $n+n'$ qubits. Or we can say we have added an $n'$-qubit SES register to the original *n*-qubit register. If we denote the computational subspace of an *n*-qubit SES register by $\mathbb {C}^n$ the direct sum implements,

1 $$\begin{aligned} \mathbb {C}^{n} \oplus \mathbb {C}^{n'} = \mathbb {C}^{n+n'} \! \! . \end{aligned}$$

The tensor product option comes from the standard gate model of quantum computation, where each physical qubit contributes a two-dimensional complex Hilbert space $\mathbb {C}^2$, and the computational Hilbert space of *n* qubits is the tensor product $\mathbb {C}^2 \otimes \mathbb {C}^2 \otimes \cdots \otimes \mathbb {C}^2 = \mathbb {C}^{2^n}$. In this paper we implement a tensor product of the form

2 $$\begin{aligned} \mathbb {C}^{n} \otimes \mathbb {C}^2, \end{aligned}$$

which adds an excitation and is equivalent to $\mathbb {C}^{2n}.$ The added qubit can be used as an ancilla to control the application of arbitrary unitaries to the data $|\psi \rangle$ stored in $\mathbb {C}^{n}$, enabling transformations from product states

3 $$\begin{aligned} |\psi \rangle \otimes \left( \alpha |0\rangle _{n+1} + \beta |1\rangle _{n+1} \right) \end{aligned}$$

to arbitrary states of the form

4 $$\begin{aligned} \alpha \, \left( U_0 |\psi \rangle \right) \otimes |0\rangle _{n+1} + \beta \, \left( U_1 |\psi \rangle \right) \otimes |1\rangle _{n+1}. \end{aligned}$$

here $|0\rangle _{n+1}$ and $|1\rangle _{n+1}$ are states of qubit $n+1$, the ancilla (not a register of $n+1$ qubits). Adding $n'$ ancilla in this manner implements

5 $$\begin{aligned} \mathbb {C}^{n} \otimes \underbrace{ \mathbb {C}^2 \otimes \cdots \otimes \mathbb {C}^2}_{n' \ {\text{ qubits }}} = \mathbb {C}^{n} \otimes \mathbb {C}^{2^{n'}} = \mathbb {C}^{n 2^{n'}} \! \! . \end{aligned}$$

An example is given in Fig. 1b. Because the computational subspace (5) is exponential in $n'$, larger problem sizes become possible. We propose this extension of the SES method as a possible route to practical quantum computation with NISQ technology.

## Controlled-unitary protocol

### SES computer chip

**Figure 2:**
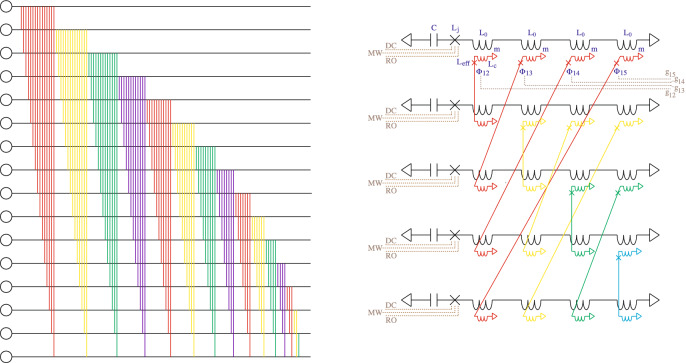
Possible layout for an SES chip. On the left, the circles represent qubits, the horizontal lines are wires, and the colored vertical lines are couplers. This is shown in more detail on the right for $n\!=\!5$. Each horizontal circuit is a superconducting qubit with capacitance *C*, tunable junction inductance $L_{\text{ j }}$, and $n-1$ additional coils (each with self-inductance $L_0$ and mutual inductance *m*) for coupling to other qubits. Brown dotted lines indicate dc and microwave control lines for each qubit, as well as readout circuits. Each colored coupler wire contains a Josephson junction with inductance $L_{\text{ c }}$ tuned by a magnetic flux $\Phi$. Control lines for some off-diagonal SES Hamiltonian matrix elements are also indicated in brown.

The hardware required for ancilla-assisted SES computation is identical to that described in Geller et al.^6^ i.e., a complete graph of superconducting transmon^8^ or Xmon^9^ qubits with tunable frequencies and tunable $\sigma ^x \otimes \sigma ^x$ couplings^10^. The device Hamiltonian is

6 $$\begin{aligned} H_{\text{ qc }} = \sum _{i} \begin{pmatrix} 0 &{}\quad 0 \\ 0 &{}\quad \epsilon _i \end{pmatrix}_{\! \! i} + \frac{1}{2} \sum _{i i'} g_{ii'} \, \sigma ^x_i\otimes \sigma ^x_{i'}, \end{aligned}$$

with $\epsilon _i$ and $g_{ii'}$ tunable. $g_{ii'}$ is a real, symmetric matrix with vanishing diagonal elements. A possible chip layout is shown in Fig. 2.

### SES method basics

In the SES method without ancillas^6,7^, computations are performed in the *n*-dimensional subspace spanned by the basis states

7 $$\begin{aligned} |i) := |0 \cdots 1_i \cdots 0\rangle , \ \ \ i \in \lbrace 1, 2, \ldots , n \rbrace . \end{aligned}$$

A pure state in the SES has the form

8 $$\begin{aligned} |\psi \rangle = \sum _{i=1}^n a_i \, |i), \ \ \ {\text{ with }} \ \ \ \sum _{i=1}^n |a_i|^2 = 1. \end{aligned}$$

The advantage of working in the SES is that the matrix elements

9 $$\begin{aligned} \mathcal{H}_{ii'} := ( i | H_{\text{qc}} | i' ) = \epsilon _i \, \delta _{ii'} + g_{ii'} \end{aligned}$$

of (6) can be directly controlled. *We can therefore directly program the Hamiltonian of the computer chip.*

The protocol for implementing a specific operation depends on the functionality (available ranges of the $\epsilon _i$ and $g_{ii'}$) of the SES chip. In this paper we assume that the experimentally controlled SES Hamiltonian can be written, apart from an additive constant, in the *standard form*

10 $$\begin{aligned} \mathcal{H} = g_{\text{max}} K \quad {\text{ with }} \quad -1 \le K_{i i'} \le 1. \end{aligned}$$

Here $g_{\text{max}}$ is the maximum interaction strength provided by the coupler circuits. A reasonable value for $g_{\text{max}}/h$ is 10–$50 \, {\text{ MHz }}$.

The basic single-step operation in SES quantum computing is the application of a symmetric unitary of the form $e^{-iA}$ to the data, where *A* is a given real symmetric matrix. If only the unitary $e^{-iA}$ is known, the classical overhead for obtaining *A* from $e^{-iA}$ is to be included in the quantum runtime. (Note that the generator *A* is not unique because the matrix logarithm is not unique.) Define,

11 $$\begin{aligned} \theta _{\!A} := \max _{i i'} |A_{ii'} - c \delta _{ii'}|, \end{aligned}$$

where $c = (\min _{i} A_{ii}+\max _{i} A_{ii})/2$. The optimal SES Hamiltonian $\mathcal{H}$ to implement $e^{-iA}$ up to a phase is given by the standard form (10), with

12 $$\begin{aligned} K = \frac{A-cI}{\theta _{\!A}}. \end{aligned}$$

Here *I* is the $n \times n$ identity, and the matrix elements of (12) satisfy $|K_{ii'}|\le 1$. The associated evolution time is

13 $$\begin{aligned} t_{A}= \frac{\hbar \theta _{\!A}}{g_{\text{max }}}. \end{aligned}$$

Additional discussion of these results is provided in Geller et al.^6^ and Katabarwa and Geller^7^.

In an experimental implementation, then, the operation $e^{-iA}$ results from evolution under the Hamiltonian $H_{\text{qc}}$ with $\epsilon _i = \epsilon _0 + g_{\text{max }} K_{ii}$, where $\epsilon _0$ is a fixed qubit idling frequency, and $g_{i i'} = g_{\text{max}} K_{ii'} \, (i\ne i'),$ for a time duration $t_A$. It is not even necessary for $\mathcal{H}$ to be abruptly switched on and off: Any SES Hamiltonian of the form $\mathcal{H} = g(t) K$ such that $\int (g/\hbar ) \, dt = \theta _{\!A}$ may be used.

### Single-hole states

The idea underlying the controlled-unitary protocol is to use the non-SES states

14 $$\begin{aligned} {\overline{|i)}} := \left( \sigma ^x\right) ^{\! \otimes n} |i) = |1 \cdots 1 0_i 1 \cdots 1\rangle , \end{aligned}$$

which have $n-1$ excitations and which are particle-hole dual to the SES basis states. The dual state ${\overline{|i)}}$ has a single hole (absence of excitation) in qubit *i*. In a graph with $g_{ii'}=0$, the basis state |*i*) is an eigenstate with energy $\epsilon _i,$ whereas ${\overline{|i)}}$ has energy $E_n -\epsilon _i$, where

15 $$\begin{aligned} E_n = \sum _{i=1}^n \epsilon _{i} \end{aligned}$$

is the energy of the filled “band” $|1 1 \cdots 1\rangle$ of *n* excitations. Therefore, apart from a shift $E_n$, the dual states have *negative* energies; the resulting minus sign is the key to the protocol.

### Description of the protocol

First we discuss the use of a single ancilla. The objective is to implement the controlled-unitary

16 $$\begin{aligned} U \otimes |0\rangle \langle 0 |_{n+1} + I \otimes |1\rangle \langle 1 |_{n+1}, \end{aligned}$$

where *U* is an arbitrary $n \! \times \! n$ unitary matrix acting on the SES register, and *I* is the $n \! \times \! n$ identity. (This definition differs from the usual one by NOT gates on the ancilla; we assume that the additional NOT gates are included in the complete protocol.) Partition an $n+1$-qubit complete graph into an *n*-qubit SES register and one ancilla. The initial state is of the form

17 $$\begin{aligned} |\psi \rangle \otimes \left( \alpha \, |0\rangle _{n+1} + \beta \, |1\rangle _{n+1} \right) \end{aligned}$$

or

18 $$\begin{aligned} \left( \sum _{i=1}^n a_i \, |i) \right) \otimes \left( \alpha |0\rangle _{n+1} + \beta |1\rangle _{n+1} \right) . \end{aligned}$$

Next write the unitary in (16) in spectral form as $V e^{-iD} V^\dagger$, or equivalently

19 $$\begin{aligned} U = V e^{-iD/2} e^{-iD/2} V^\dagger , \end{aligned}$$

where *V* is unitary and *D* is a real diagonal matrix. We will make *U* conditional by implementing

20 $$\begin{aligned} U = V e^{-iD/2} e^{\pm iD/2} V^\dagger \end{aligned}$$

instead of (19), where the plus sign comes from the negative energy of the single-hole states and results in an application of the identity.

The first three steps of the protocol are to implement the $V^\dagger$ operation in (20) on the data stored in the SES register. The *KAK* decomposition^7^ is used to write *V* and $V^\dagger$ as

21 $$\begin{aligned} V = e^{-iA} e^{-iB} e^{iA} \ \ {\text{ and }} \ \ V^\dagger = e^{-iA} e^{iB} e^{iA}, \end{aligned}$$

where *A* and *B* are real symmetric matrices. The procedure for computing *A* and *B* is given in Katabarwa and Geller^7^. Each operator produced by the *KAK* decomposition is a symmetric unitary and can be implemented in a single step (see “SES method basics”). The first operation in the protocol, $e^{iA}$, results from evolution under $H_{\text{qc}}$ with $\epsilon _i = \epsilon _0 + g_{\text{max}} K_{ii}$ ($\epsilon _0$ is a fixed qubit idling frequency) and $g_{i \ne i'} = g_{\text{max}} K_{ii'},$ with $K=-(A-cI)/\theta _{\!A},$ for a time duration $t_A = \hbar \theta _{\!A} /g_{\text{max}}$. The indices *i* and $i'$ in these expressions include the SES partition $\lbrace 1,\ldots ,n\rbrace$ only, and during this operation all couplings to the ancilla are turned off ($g_{i,n+1}=0$ for all $i \in \lbrace 1,\ldots ,n\rbrace ).$ These settings program the SES Hamiltonian $\mathcal{H} = g_{\text{max}} K$ into the chip. The ancilla qubit frequency $\epsilon _{n+1}$ is set to $\epsilon _0$. The protocol implements the symmetric unitary $e^{iA}$ up to a phase, with that phase chosen to minimize the operation time $t_{\text{ A }}$. $e^{-iA}$ is implemented by changing $K \rightarrow -K$. $e^{\pm iB}$ are implemented by changing $A \rightarrow B$. The total time required to implement $V^\dagger$ is $t_{V} \! = \! 2t_{A} + t_{B}$. After these steps (18) becomes

22 $$\begin{aligned} \left( \sum _{i=1}^n (V^\dagger a)_i \, |i) \right) \otimes \left( \alpha |0\rangle _{n+1} + e^{-i \epsilon _0 t_{V}/\hbar } \beta |1\rangle _{n+1} \right) , \end{aligned}$$

where, for any unitary *W* acting on the SES, we write $\sum _{i'} (i|W|i') \, a_{i'}$ as $(Wa)_i$. Note that the ancilla acquired a relative phase after these operations; we assume that such phases are removed by applying *z* rotations to the ancilla or by working in a rotating frame.

The next steps apply $e^{\pm i D/2}$ conditioned on the ancilla, the sign change resulting from the negative energies of the dual states. After CNOT gates between the ancilla (control) and each of the *n* SES qubits (targets), we have

23 $$\begin{aligned} \sum _{i=1}^n \left( \! (V^\dagger a)_i \, |i) \otimes \alpha \, |0\rangle _{n+1} + (V^\dagger a)_i \, {\overline{|i)}} \otimes \beta \, |1\rangle _{n+1} \! \right) . \end{aligned}$$

In “Multi-target CNOT” we show how to implement these *n* CNOT gates *simultaneously*. Then follow the protocol as if to implement the diagonal operator $e^{-iD/2}$ in the SES: apply $H_{\text{qc}}$ with $g_{i i'}\!=\!0$ and $\epsilon _i = \epsilon _0 + g_{\text{max }} K_{ii}$ for a time $t_D = \hbar \theta _{\!D} / 2 g_{\text{max }}$. Here $K \!:=\! (D - cI)/\theta _{\!D},$ $\theta _{\!D} \! := \! \max _{i} |D_{ii}-c|$, and $c = (\min _{i} D_{ii}+\max _{i} D_{ii})/2.$ Set the ancilla frequency to $\epsilon _0.$ Following this operation, and another ancilla *z* rotation (23) becomes

$$\begin{aligned} \sum _{i=1}^n \left( (e^{-iD/2} V^\dagger a)_i \, |i) \otimes \alpha \, |0\rangle _{n+1} + ( e^{iD/2} V^\dagger a)_i \, {\overline{|i)}} \otimes \beta \, |1\rangle _{n+1} \right) . \end{aligned}$$

**Figure 3:**
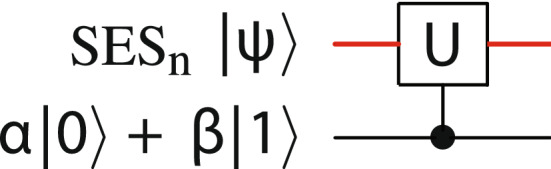
Controlled unitary operation. The red upper line is an *n*-qubit SES register, the lower line is the ancilla qubit.

After a second set of CNOT gates and subsequent application of $e^{-iD/2}$ and *V* to the SES, and a final ancilla *z* rotation, we obtain

24 $$\begin{aligned} \sum _{i=1}^n \left( (Ua)_i \, |i) \otimes \alpha \, |0\rangle _{n+1} + a_i \, |i)\otimes \beta \, |1\rangle _{n+1} \right) , \end{aligned}$$

or

25 $$\begin{aligned} U |\psi \rangle \otimes \alpha \, |0\rangle _{n+1} + |\psi \rangle \otimes \beta \, |1\rangle _{n+1}, \end{aligned}$$

as required. We represent this operation—including implicit NOT gates on the ancilla before and after transformation (16)—by the circuit diagram of Fig. 3.

We have described the use of a single ancilla qubit. The total number of steps required to implement the controlled unitary (about 10) is independent of *n*. Additional ancilla can be included by increasing the graph size by one for each new ancilla. Each ancilla independently controls unitaries acting on the shared data register. These unitaries, however, cannot be performed simultaneously, so in most applications the runtime will scale linearly with the number of ancilla $n'\!.$

**Figure 4:**
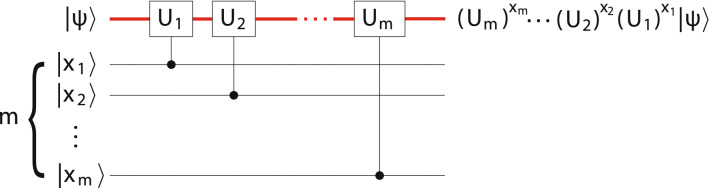
A circuit with *m* distinct controlled unitaries in succession. The red upper line is an *n*-qubit SES register. When the *m* ancilla are in a computational basis state $| x_1 x_2 \cdots x_m\rangle$ with $x_i \in \lbrace 0, 1\rbrace$, the action on the SES register is $| \psi \rangle \mapsto (U_m)^{x_m} \cdots (U_2)^{x_2} (U_1)^{x_1}| \psi \rangle$.

As an example, in Fig. 4 we consider a quantum circuit that implements *m* controlled unitaries $U_1, U_2, \ldots , U_m$ in succession, each controlled by a single ancilla. This circuit applies the operator

26 $$\begin{aligned} \sum _{x=0}^{{2^m}-1} (U_m)^{x_m} \cdots (U_2)^{x_2} (U_1)^{x_1} \otimes |x\rangle \langle x | \end{aligned}$$

to the computational Hilbert space, where $x_j$ is the *j*th bit in the binary representation for *x*,

27 $$\begin{aligned} x = \sum _{j=1}^m 2^{m-j} x_j. \end{aligned}$$

A common special case of (26) is to let the $U_j = (U)^{2^{m-j}}$ for some unitary *U*, in which case the circuit of Fig. 4 implements the operation

28 $$\begin{aligned} \sum _{x=0}^{{2^m}-1} U^x \otimes |x\rangle \langle x |. \end{aligned}$$

### Multi-target CNOT

The protocol of the last section requires two rounds of CNOT gates applied between the ancilla (control) and the *n* qubits in the SES partition (targets). For small *n* these can be done serially using the high-fidelity entangling gates developed for standard gate-based superconducting quantum computation. The CNOT gates commute, however, and in principal can be performed simultaneously. Multi-target CNOT gate protocols^11–18^ have been developed for ion trap, cavity QED, and circuit QED architectures, where many qubits can be coupled to a common cavity or other bosonic mode. These protocols can be applied to the complete graph architecture as well, but at the expense of supplementing each ancilla with an additional resonator or qubit, which must also be fully connected. The desired tensor product would then require $n+2n'$ qubits. We avoid this overhead by designing a fast multi-target CNOT gate specifically for the complete graph.

It is well known that the entangling gate

29 $$\begin{aligned} e^{-i \frac{\pi }{4} \sigma ^x \otimes \sigma ^x} \end{aligned}$$

is locally equivalent to a two-qubit CNOT gate, meaning that it is a CNOT apart from single-qubit rotations. To see this, let the second qubit be the control, and apply Hadamards to obtain $e^{-i \frac{\pi }{4} \sigma ^x \otimes \sigma ^z} \! \!.$ This operator acts with $e^{- i \frac{\pi }{4} \sigma ^x}$ on the target when the control is $|0\rangle$, and with $e^{i \frac{\pi }{4} \sigma ^x}$ when the control is $|1\rangle$, from which it is straightforward to construct a CNOT.

**Figure 5:**
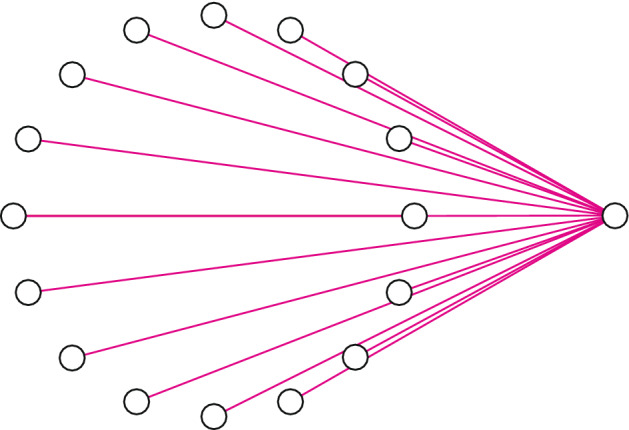
Graph to implement the *n*-target CNOT gate.

It is not surprising that a simultaneous multi-target CNOT gate is possible in the complete graph architecture, and the Hamiltonian (6) already contains an interaction underlying such an operation: set the couplings between the ancilla and the *n* qubits in the SES partition to a positive constant *g* and all others to zero, as illustrated in Fig. 5. The interaction

30 $$\begin{aligned} g \left( \sum _{i=1}^n \sigma ^x_i \right) \otimes \sigma ^x_{n+1} \end{aligned}$$

couples the ancilla qubit to a collective variable

31 $$\begin{aligned} S_x = \sum _{i=1}^n \sigma ^x_i \end{aligned}$$

of the SES partition. Such an interaction, *on its own*, can be used to generate the desired multi-qubit entangling operation

32 $$\begin{aligned} e^{-i \frac{\pi }{4} S_x \otimes \sigma ^x_{n+1}} = \prod _{i=1}^n e^{-i \frac{\pi }{4} \sigma ^x_i \otimes \sigma ^x_{n+1}} \end{aligned}$$

that generalizes (29). The multi-target CNOT gate

33 $$\begin{aligned} I \otimes |0\rangle \langle 0 |_{n+1} + (\sigma ^x)^{\otimes n} \otimes |1\rangle \langle 1|_{n+1} \end{aligned}$$

results from the gate sequence

34 $$\begin{aligned} \begin{pmatrix} 1 &{}\quad 0 \\ 0 &{}\quad -i \\ \end{pmatrix}_{\! n+1} \! \! \! (e^{i \frac{\pi }{4} \sigma ^x} )^{\otimes n} \, \mathsf{H}_{n+1} \ e^{-i \frac{\pi }{4} S_x \otimes \sigma ^x_{n+1}} \ \mathsf{H}_{n+1} , \end{aligned}$$

where $\mathsf{H}$ is the single-qubit Hadamard gate.

The device Hamiltonian (6), however, contains single-qubit terms that do not commute with (30). Therefore it will be necessary follow a modified protocol to obtain the entangler (32): add a $\sigma ^x$ microwave drive to the ancilla and transform to the usual rotating frame, where the $\sigma ^x \otimes \sigma ^x$ interaction becomes $\frac{1}{2}(\sigma ^x \otimes \sigma ^x + \sigma ^y \otimes \sigma ^y)$, and then transform to a second rotating frame where the interaction is $\frac{1}{2} \sigma ^x \otimes \sigma ^x.$ This is discussed further in the “Supplementary Information”.

Finally, we discuss the expected performance of this design when implemented in a transmon-based chip with inductive couplers. The main source of error is leakage into higher lying $|2\rangle$ states neglected in (6) but present in a real device. Although the current design has not been optimized to minimize this leakage, the estimated performance is already satisfactory for initial demonstrations, as indicated in Table 1.

**Table 1:** Performance of simultaneous *n*-target CNOT gate in complete graph of $n\!+\!1$ transmon or Xmon qubits, using realistic models for the qubits and couplers.

*n*	$\eta /h ({\text{ MHz }})$	$t_{\text{gate}} ({\text{ ns }})$	$\Omega /h ({\text{ MHz }})$	$g/h ({\text{ MHz }})$	$E_{\text{gate}}$ %
3	300	40	100	6.25	1.2
4	300	40	150	6.25	1.7
5	300	40	150	6.25	2.4

Here $\eta$ is the qubit anharmonicity, $t_{\text{gate}}$ is the gate time excluding the single-qubit rotations in (34), $\Omega$ is the microwave Rabi frequency, and *g* is the coupler strength. The reported gate error is $E_{\text{gate}} = 1 - |\langle \Psi | U_{\text{ ideal }} ^\dagger U | \Psi \rangle |^2 \! ,$ where $U_{\text{ideal}}$ is the ideal entangler (32), and *U* is the realized evolution operator computed in the absence of decoherence. The error is averaged over initial states $|\Psi \rangle$. The qubit frequencies are $\epsilon _0/h = 5.5\,{ \text{ GHz }}.$

## Applications

### Phase estimation

Energy estimation via quantum phase estimation is a natural application of the SES method and has been discussed in detail in Geller et al.^6^ where it was explained that the Hamiltonian simulation component can be done exactly without Trotter errors. This is because one can directly program the individual matrix elements of the Hamiltonian. But that implementation requires 2*N* qubits to simulate a $N \times N$ Hamiltonian. Using the controlled-unitary protocol introduced here, however, allows the same algorithm to be implemented with a complete graph of $N+1$ qubits. The main ingredients of the implementation, including adiabatic state preparation, have already been discussed^6^ and will not be repeated here.

### Matrix inversion

As a second application of SES fusion we give an ancilla-assisted implementation of the quantum linear system solver of Harrow, Hassidim, and Lloyd^19^. We do not expect an SES chip running this implementation to outperform a classical supercomputer. We choose this algorithm because it requires a large register of ancilla qubits, which is challenging, and because it has interesting generalizations and applications to machine learning.

The matrix inversion algorithm^19,20^ solves the linear system $A \mathbf{x} = \mathbf{b}$ for $\mathbf{x}$, accepting $\mathbf{b}$ in the form of a normalized pure state $|\mathbf{b} \rangle$, and returning the solution in the form of a pure state $|\mathbf{x} \rangle$. In the SES implementation these states are stored in a data register of Hilbert space dimension *n*. (Note that in our notation *A* is $n\!\times \!n,$ not $2^n\!\times \!2^n$.) A second register of *m* qubits is used for the phase estimation subroutine, and one more is used for postselection. The value of *m* determines the accuracy of the solution. The SES implementation (for symmetric *A*) requires a complete graph of $n+m+1$ qubits.

**Figure 6:**
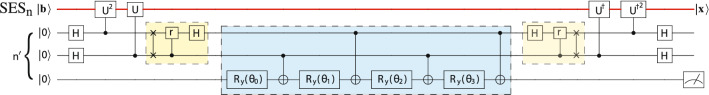
Quantum circuit for $n\! \times \! n$ matrix inversion with $m=2$. Here $\mathsf{H}$ is the Hadamard gate, $U = e^{i A t_0/2^m} \! \! ,$ the vertical line connecting crosses is a $\mathsf{SWAP}$ gate, $r = |0\rangle \langle 0| + i |1\rangle \langle 1|$ is a *z* rotation, and $R_y = e^{-i(\theta /2) \sigma ^y}$ is a *y* rotation. The small (yellow) subcircuits are Fourier transforms and the central (blue) subcircuit implements the controlled ancilla rotation (35).

The circuit for $m=2$ is given in Fig. 6. The central (blue) subcircuit implements the controlled-rotation operation

35 $$\begin{aligned} \sum _{k=0}^{2^m-1} |k\rangle \langle k | \otimes R_y(\gamma _k), \ \ \gamma _{k} := {\left\{ \begin{array}{ll} 2 \arcsin \left( \frac{1}{k} \right) &{} {\text {for }} k > 0,\\ 0 &{} {\text {for }} k = 0. \end{array}\right. } \end{aligned}$$

here $|k\rangle$ is a computational basis state of the m-qubit ancilla register. The rotation angles $\theta _0 , \ldots , \theta _3$ in Fig. 6 are determined by finding the net *y* rotation applied to the last qubit in each of the cases $|k\rangle \in \lbrace |00\rangle , |01\rangle , |10\rangle , |11\rangle \rbrace$, making use of the identity $\sigma ^x \, R_y(\theta ) \, \sigma ^x = R_y(-\theta ),$ and comparing the result with (35), rewritten as

36 $$\begin{aligned}&|00\rangle \langle 00| \otimes R_y(\gamma _0) + |01\rangle \langle 01| \otimes R_y(\gamma _1) \nonumber \\&\quad + |10\rangle \langle 10| \otimes R_y(\gamma _2) + |11\rangle \langle 11| \otimes R_y(\gamma _3). \end{aligned}$$

This leads to

37 $$\begin{aligned} \begin{pmatrix} 1 &{}\quad 1 &{}\quad 1 &{}\quad 1 \\ 1 &{}\quad -1 &{}\quad -1 &{}\quad 1 \\ 1 &{}\quad 1 &{}\quad -1 &{}\quad -1 \\ 1 &{}\quad -1 &{}\quad 1 &{}\quad -1 \end{pmatrix} \begin{pmatrix} \theta _0 \\ \theta _1 \\ \theta _2 \\ \theta _3 \end{pmatrix} = \begin{pmatrix} \gamma _0 \\ \gamma _1 \\ \gamma _2 \\ \gamma _3 \end{pmatrix} \! , \end{aligned}$$

with the $\gamma _k$ given in (35). The matrix in (37), after multiplication by $2^{-m/2}$, is orthogonal and hence immediately inverted, yielding the $\theta _k$.

**Figure 7:**
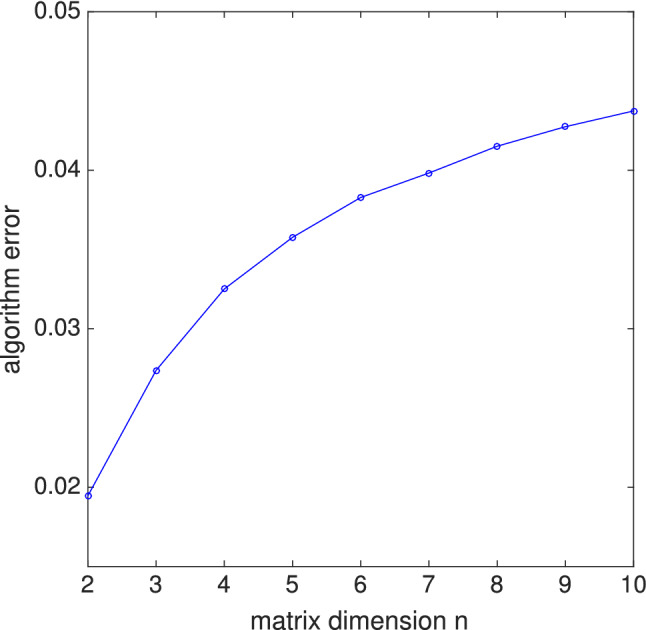
Matrix inversion algorithm error for $m=3$, averaged over random real symmetric matrices *A* (with eigenvalues $0< \lambda _i < 1$). The error computed here results from the low precision of the phase estimation procedure (small *m* value) only and does not include the effects of decoherence and other errors that would arise during implementation.

The $m=2$ phase estimation is not sufficiently accurate for matrix inversion, typically leading to 5–15% algorithm errors for matrix sizes $2 \le n \le 4.$ By algorithm error we mean

38 $$\begin{aligned} E_{\text{alg}} = 1 - \langle \mathbf{x}_{\text{ ideal }} | \rho _{\text{data}} | \mathbf{x}_{\text{ ideal }} \rangle , \end{aligned}$$

where $\rho _{\text{data}}$ is the final state of the data register, traced over the $m+1$ ancilla, and $| \mathbf{x}_{\text{ideal }} \rangle$ is the pure state corresponding to the exact solution of the given linear system. The circuit of Fig. 6 can be easily extended to larger *m*, however, and the performance for $m=3$ is already sufficient for an initial demonstration. The controlled-rotation subcircuit for $m > 2$ can be obtained from the “uniformly controlled” rotation operator construction of Möttönen et al.^21^ which requires $2^m$ CNOT gates (and is therefore not useful for large *m*). Simulating the $m=3$ circuit we find that real symmetric matrices up to dimension 10 can be inverted with algorithm errors less than 5%, as shown in Fig. 7, a considerable increase in problem size over the existing gate-based realizations^22–26^.

## Conclusions

In this paper we have introduced a non-gate-model form of NISQ computation that extends the reach of the standard SES method^6,7^ by allowing the use of ancilla as control qubits, without doubling the graph size for each ancilla. This should make it possible to apply the SES method to larger problem sizes. We propose this method as a route to practical quantum computation with NISQ technology.

While it is interesting to discuss the SES method and other NISQ approaches in terms of their runtime complexity, this can be misleading because they are not scalable. For example the SES method allows one to implement arbitrary $n\times n$ unitaries in constant time^7^, which even a fault-tolerant quantum computer cannot do efficiently. However this ignores the fact that decoherence and other errors will limit the largest problems sizes that can be reliably implemented. But the constant runtime does suggest that it could be a good starting point for developing near-term applications with a quantum advantage.

## Supplementary information

Supplementary Information.

## Data Availability

Simulation data is available upon request.
