# Supplementary material for: Fusing the single-excitation subspace with ℂ2n
Source: Sci Rep. 2021 Jan 11;11:402. doi: 10.1038/s41598-020-79853-3 (PMC7801700; doi:10.1038/s41598-020-79853-3)
Supplement: Supplementary file 1 — Supplementary Information. [file 41598_2020_79853_MOESM1_ESM.pdf]

# Supplementary Information for "Fusing the single-excitation subspace with $\mathbb{C}^{2^n}$"

Michael R. Geller[1]

[1]*Center for Simulational Physics, University of Georgia, Athens, Georgia 30602 USA*

## I. MULTI-QUBIT ENTANGLER DESIGN

Here we show how to produce the entangler (32) used to construct the multi-target CNOT gate. With the couplings between the ancilla and the $n$ qubits in the SES partition set to a positive constant $g$ (see Fig. 5), the device Hamiltonian becomes

$$H = \sum_{i=1}^{n+1} \begin{pmatrix} 0 & 0 \\ 0 & \epsilon_0 \end{pmatrix}_i + g \sum_{i=1}^{n} \sigma_i^x \otimes \sigma_{n+1}^x + \Omega \cos(\tfrac{\epsilon_0 t}{\hbar})\, \sigma_{n+1}^x, \quad (1)$$

where we have added a resonant microwave drive to the ancilla. Decompose the time evolution operator $U$ generated by (**??**) as

$$U = e^{-iD_a t/\hbar} U_a, \quad \text{with} \quad D_a = \sum_{i=1}^{n+1} \begin{pmatrix} 0 & 0 \\ 0 & \epsilon_0 \end{pmatrix}_i. \quad (2)$$

Then $\dot{U}_a = -(i/\hbar) H_a U_a$, where

$$H_a \approx \frac{g}{2} \sum_{i=1}^{n} \Big( \sigma_i^x \otimes \sigma_{n+1}^x + \sigma_i^y \otimes \sigma_{n+1}^y \Big) + \frac{\Omega}{2} \sigma_{n+1}^x \quad (3)$$

is the Hamiltonian in the $z$-rotating frame. We choose the evolution time to satisfy

$$t_{\rm gate} = l_a \bigg( \frac{2\pi\hbar}{\epsilon_0} \bigg), \quad (4)$$

where $l_a$ is an integer, which makes $e^{-iD_a t/\hbar} = I$, and which also suppresses the small corrections to (**??**) when averaged over $t_{\rm gate}$. The value of $l_a$ (usually between 100 and 300) is determined by the desired gate time.

Next decompose $U_a$ as

$$U_a = e^{-iD_b t/\hbar} U_b, \quad \text{with} \quad D_b = \frac{\Omega}{2} \sigma_{n+1}^x. \quad (5)$$

Then $\dot{U}_b = -(i/\hbar) H_b U_b$, where

$$H_b \approx \frac{g}{2} \sum_{i=1}^{n} \sigma_i^x \otimes \sigma_{n+1}^x = \frac{g}{2} S_x \otimes \sigma_{n+1}^x \quad (6)$$

is the Hamiltonian in a second frame rotating the ancilla about the $x$ axis. We choose the Rabi frequency to satisfy

$$\Omega = l_b \bigg( \frac{4\pi\hbar}{t_{\rm gate}} \bigg), \quad (7)$$

where $l_b$ is another integer, which makes the corrections to (**??**) vanish on average and also makes $e^{-iD_b t/\hbar} = \pm I$. The value of $l_b$ is chosen to minimize the total gate error: For the simulations reported in Table I we find that $l_b = 2$ or 3 is optimal. The effect of this second transformation is to remove the $\sigma^y \otimes \sigma^y$ term in (**??**). Note the factor of $\frac{1}{2}$ in (**??**) that is not present in (**??**).

With these parameters the evolution operator becomes

$$U \approx e^{-i \frac{g}{2} S_x \otimes \sigma_{n+1}^x t_{\rm gate}}. \quad (8)$$

Setting the coupling strength to $g = \pi\hbar/2t_{\rm gate}$ generates the desired entangler (32).
